# The concentrated antibody from convalescent plasma balanced the dysfunctional immune responses in patients with critical COVID‐19

**DOI:** 10.1002/ctm2.571

**Published:** 2021-11-04

**Authors:** Mingfeng Liao, Xuejiao Liao, Jing Yuan, Bing Zhou, Yang Liu, Xiao Jiang, Zhan Zhang, Caiping Guo, Yunjia Zhang, Shuye Zhang, Lei Liu, Zheng Zhang

**Affiliations:** ^1^ National Clinical Research Center for Infectious Disease Institute for Hepatology Shenzhen Third People's Hospital The Second Affiliated Hospital School of Medicine Southern University of Science and Technology Shenzhen Guangdong Province China; ^2^ Department for Infectious Diseases Shenzhen Third People's Hospital Shenzhen China; ^3^ Guangdong Province Research & Development Centre of Protein (Peptide) Separation Engineering Technology Shenzhen China; ^4^ Shanghai Public Health Clinical Center Fudan University Shanghai China; ^5^ Shenzhen Research Center for Communicable Disease Diagnosis and Treatment of Chinese Academy of Medical Science Shenzhen Guangdong Province China; ^6^ Shenzhen Bay Laboratory Shenzhen Guangdong Province China

Dear Editor,

This study found that the concentrated antibody therapy was able to clear residual virus reservoirs, increase neutralizing antibody levels and the overall immune response by enhancing peripheral lymphocyte counts and neutrophil–lymphocyte ratio when given during the early phase of SARS‐CoV‐2 infection.

**To the Editor**:

Due to the absence of specific and effective treatment for coronavirus disease‐2019 (COVID‐19), antibody therapy is of great interest.^1^ With the strong specificity, the antibody therapy is suitable for emergency use. While therapeutic antibodies and convalescent plasma were promising and facilitating patient recovery,^2^, ^3^, ^4^, ^5^ the clinical benefit and immunological effects of therapies using plasma and concentrated antibody from convalescent donors remain uncertain. Here, we conducted a non‐randomized clinical trial using concentrated antibodies to treat critically ill COVID‐19 patients.

Thirteen COVID‐19 patients were enrolled in this study at the Shenzhen Third People's Hospital from late January to August, 2020. The demographic data of the patients were shown in Table 1. Seven patients (S1–S7) received concentrated antibody and (or) convalescent plasma treatment as the intervention group, and the other six patients (S8–S13) without no plasma or antibody therapy were followed as the control group. Health history of the both groups showed a very slight and nonsignificant difference (Table S1). Concentrated antibodies were derived from the plasma of fourteen convalescent COVID‐19 donors (Table 2, Supplemental Methods and Materials).

**TABLE 1:** Clinical characteristics of 13 COVID‐19 patients at Shenzhen third people's hospital, January to August 2020

	S1	S2	S3	S4	S5	S6	S7	S8	S9	S10	S11	S12	S13
Severity	Critical	Critical	Critical	Critical	Critical	Critical	Critical	Critical	Critical	Critical	Critical	Critical	Critical
Age (years)	46	73	69	64	69	70	29	72	36	69	57	66	63
Gender	Male	Male	Male	Female	Male	Female	Male	Male	Male	Male	Female	Male	Female
Wuhan traveling	Yes	Yes	Yes	No	No	No	No	Yes	No	Yes	No	No	No
Symptom onset date/first symptom	2020‐1‐21 Fever, cough	2020‐1‐20 Fever	2020‐1‐21 Fever, dizzy	2020‐1‐29 Fever, cough	2020‐1‐31 Fever	2020‐3‐4 Fever	2020‐4‐24 Fever	2020‐1‐4 Fever, cough	2020‐1‐19 Fever, cough	2020‐1‐11 Fever, cough, pharyngeal pain	2020‐1‐21 Dizzy, cough, pharyngeal pain	2020‐1‐20 Fever, weak	2020‐1‐12 Fever, cough
Hospitalization date	2020‐1‐22	2020‐1‐22	2020‐1‐24	2020‐2‐7	2020‐2‐3	2020‐3‐6	2020‐4‐27	2020‐1‐20	2020‐1‐28	2020‐1‐20	2020‐1‐23	2020‐1‐24	2020‐1‐27
Convalescent Plasma Transfusion (date)	ND	Yes 2020‐2‐13; 2020‐2‐23	ND	ND	Yes 2020‐2‐15	ND	Yes 2020‐5‐3	ND	ND	ND	ND	ND	ND
Neutralizing antibody Transfusion (date)	Yes 2020‐2‐2	Yes 2020‐2‐2; 2020‐3‐7	Yes 2020‐2‐19	Yes 2020‐2‐19	Yes 2020‐2‐19 2020‐3‐7	Yes 2020‐3‐12	Yes 2020‐5‐1	ND	ND	ND	ND	ND	ND
Outcome/date	Cured 2020‐3‐8	Death 2020‐7‐23	Death 2020‐2‐26	Cured 2020‐3‐11	Death 2020‐10‐10	Cured 2020‐4‐1	Cured 2020‐5‐21	Cured 2020‐2‐23	Cured 2020‐2‐26	Cured 2020‐2‐8	Cured 2020‐3‐7	Cured 2020‐3‐6	Cured 2020‐3‐6
Chronic basic disease	None	HTN	Diabetes	SP	HTN	Coronary heart disease	Diabetes	HTN	None	HTN	None	None	None
Medication history	None	None	None	None	None	None	None	None	None	None	None	None	None
SARS‐CoV‐2 RT‐qPCR (Ct value, Before or Transfusion Day)/sample	+(30.97) BALF 2020‐2‐2	+(27.67) Throat swab 2020‐2‐2	+(36.2) Sputum 2020‐2‐19	+(37.8) Nasal swab 2020‐02‐19	+(27.7) Sputum 2020‐2‐19	+(26.3) Sputum 2020‐3‐12	+(40) Nasal swab 2020‐4‐28	+(21.6) Sputum 2020‐1‐28	+(24.8) Nasal swab 2020‐2‐10	+(23) Sputum 2020‐1‐20	+(22.5) Throat swab 2020‐1‐24	+(26.5) Throat swab 2020‐1‐24	+(23.8) Nasal swab 2020‐1‐30
Flu A/B	–/–	–/–	–/–	–/–	–/–	–/–	–/–	–/–	–/–	–/–	–/–	–/–	–/–
RSV virus	–	–	–	–	–	–	–	–	–	–	–	–	–
Adenovirus	–	–	–	–	–	–	–	–	–	–	–	–	–
Interferon atomization	2020‐1‐22	2020‐1‐22	2020‐1‐24	2020‐2‐7	2020‐2‐4	2020‐3‐6	2020‐4‐27	2020‐1‐20	2020‐1‐28	2020‐1‐20	2020‐1‐23	No	No
Ribavirin	2020‐1‐23	2020‐1‐22	NO	2020‐2‐12	2020‐2‐11	No	No	2020‐1‐20	No	2020‐1‐20	No	No	No
Methylprednisolone	Yes	Yes	Yes	Yes	Yes	No	Yes	Yes	Yes	No	Yes	Yes	Yes

SARS‐CoV‐2, severe acute respiratory syndrome coronavirus 2; RT‐PCR, reverse transcription polymerase chain reaction; Ct, cycle threshold; BALF, bronchoalveolar lavage fluid; HTN, hypertension; SP, schizophrenic; Flu, influenza.

**TABLE 2:** Characteristics and antibody titer of convalescent plasma donors

Number	Gender	Age	Disease severity	Interval between symptom onset and discharge	Interval between discharge and plasma donation	Donated plasma volume (ml)	Blood type	For antibody therapy	For convalescent plasma	RBD‐specific IgG ELISA titer	RBD‐specific IgM ELISA titer	Neutralizing antibody titer	Infectious pathogen
1	Male	36	Moderate	17	4	500	B+	Yes	–	200	1800	NT	–
2	Male	35	Moderate	15	8	500	O+	Yes	–	600	600	NT	–
3	Male	51	Moderate	18	11	300	O+	Yes	–	278 612	670	NT	–
4	Female	52	Moderate	18	9	500	O+	Yes	–	NT	NT	NT	–
5	Male	42	Moderate	16	14	500	O+	Yes	–	259 418	171 002	NT	–
6	Male	51	Moderate	24	5	500	A+	Yes	–	16 200	48 600	NT	–
7	Male	19	Moderate	22	5	500	A+	Yes	–	16 200	48 600	NT	–
8	Female	49	Moderate	20	17	480	A+	Yes	–	112 720	6053	NT	–
9	Female	50	Moderate	19	16	400	B+	Yes	–	671 429	102 565	NT	–
10	Male	54	Moderate	16	16	400	B+	Yes	–	16 200	16 200	NT	–
11	Male	47	Severe	34	23	400	B+	Yes	–	437 400	437 400	NT	–
12	Female	36	Moderate	22	23	400	O+	Yes	–	48 600	16 200	NT	–
13	Female	47	Moderate	27	21	400	0+	Yes	–	16 200	16 200	NT	–
14	Male	28	Moderate	15	22	400	AB+	Yes	–	16 200	48 600	NT	–

RBD, receptor‐binding domain; IgG, immunoglobulin G; ELISA, enzyme‐linked immunosorbent assay.

We first investigated the effects of concentrated antibody on viral clearance. The virus Ct values began to increase after two days of concentrated antibody treatment and were then remained undetectable within 10 days posttransfusion for 6 treated patients (Figure 1A). In contrast, SARS‐COV‐2 in the control group was cleared at 14–48 days postsymptom onset (Figure S1A). The virus titer decreased significantly after concentrated antibody treatment (*P* = 0.00204) (Figure S1B).

**FIGURE 1:**
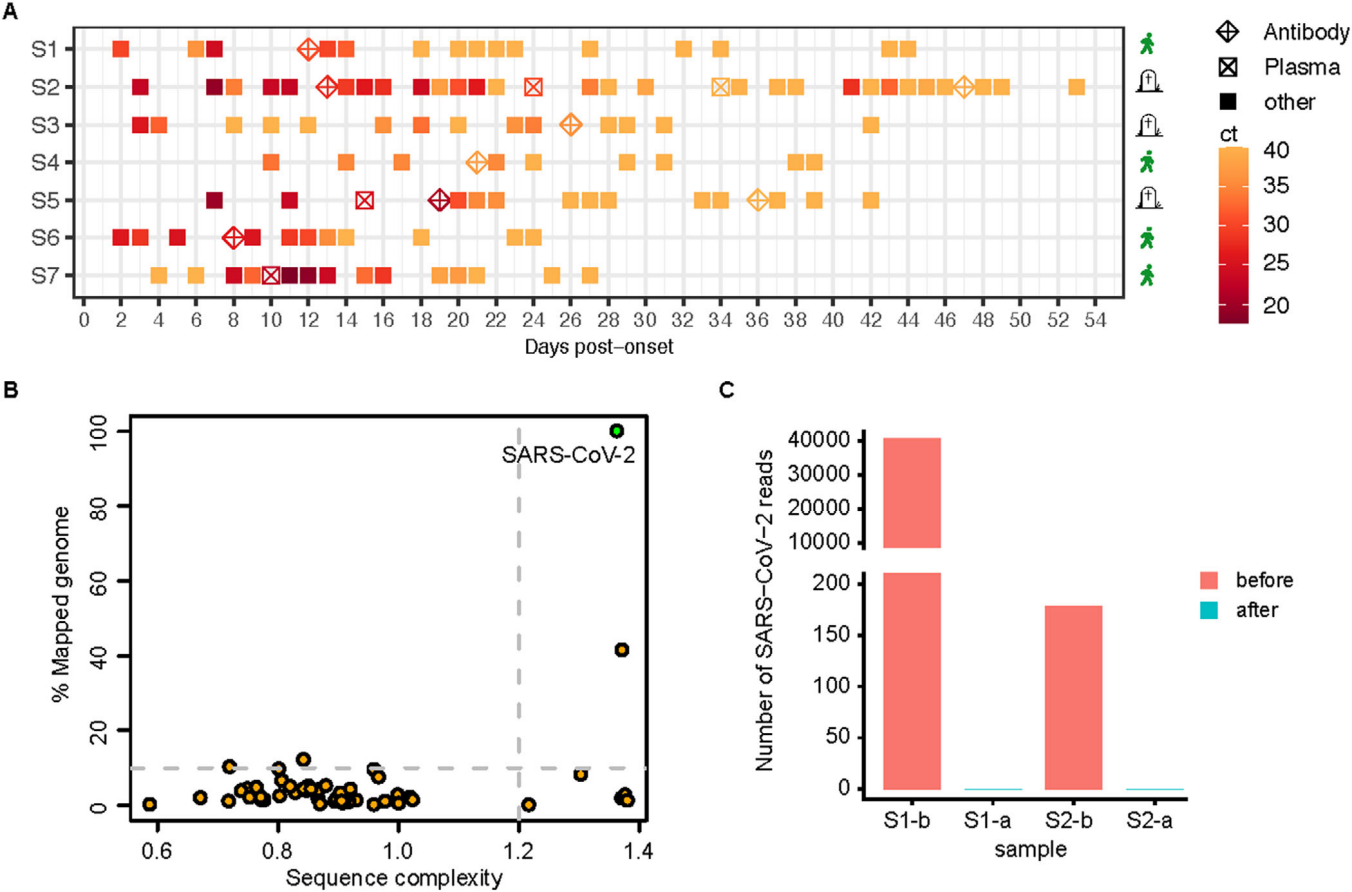
Monitoring SARS‐CoV‐2 viral loads among the enrolled COVID‐19 patients. (A) The serial viral Ct values in concentrated antibody therapy group; (B) Representative Viral‐Track analysis data. For each potential viral genome, represented by a dot, the entropy of the sequence (how repetitive are the mapped sequences) and the percentage of the segment that is mapped are plotted. Green dots correspond to viral segments that have passed quality control. Viral genomes with more than 50 mapped reads are plotted. (C) Viral‐Track analysis of SARS‐CoV‐2 reads in BALF scRNA‐seq data collected before and after the antibody treatment. D0 is the symptom onset date in (A). The square cross indicates the reception of the convalescent plasma transfusion and the diamond plus indicates the reception of concentrated neutralizing antibody treatment as indicated. Tombstone indicates fatal patients (S2, S3, S5). Pedestrian indicates cured patients (S1, S4, S6, S7)

We and others have detected residual virus in lung tissues even though continuous SARS‐CoV‐2 negativity in the nasal, throat swabs and sputum.^6^, ^7^ It was proposed that the ongoing viral activity may contribute to COVID‐19 severity. Thus, we questioned whether the concentrated antibody therapy could clear the virus reservoir. We applied Viral‐Track^8^ mining the scRNA‐seq data of Bronchoalveolar lavage fluid (BALF) samples from two patients before and after the antibody treatment. Total numbers of viral reads mapped to the SARS‐CoV‐2 viral genome were 7, 460 for S1, and 178 for S2 before the antibody treatment, respectively (Figure 1C); in contrast, no viral read was detected in samples after the treatment. We further confirmed the SARS‐CoV‐2 as the only virus in the analyzed data with Viral‐Track (Figure 1B). The viral reads were found to be enriched in the epithelial cells, plasma cells, macrophages and T cells (Figure S1C). The pathway analysis indicated an enhanced antiviral function of T cells after treatment (Figure S2).

Previous studies have reported the decreased T cell counts in peripheral blood of COVID‐19 patients; particularly, the decreased CD8 T cells were significantly correlated with disease severity.^9^ Here, we explore whether the antibody therapy affects cellular immunity. We found the concentrated antibody treatment significantly increased T cell counts in peripheral blood (Figure S3A–C). Importantly, the CD8/CD4 ratio in cured patients receiving concentrated antibody (S1, S4, S6, S7) was remained above 0.5 from 8–17 days after symptom onset, except for patient S6 whose CD8/CD4 ratio was always below 0.5 (Figure S3D; this value was the lower limitation of CD8/CD4 ratio in healthy Chinese adults). By contrast, among those patients with fatal outcomes (S2, S3, S5) even receiving concentrated antibody, the CD8/CD4 ratio decreased and reached the lowest point at 13–16 days postsymptoms onset (Figure S3E). In the control group, the CD8/CD4 ratio gradually decreased after disease onset, reaching the lowest point 14–24 days after symptom onset (Figure S3F, Extended Data 1). Neutrophil–lymphocyte ratio (NLR) was also correlated with COVID‐19 severity and prognosis, and the NLR more than 11.75 is strongly associated with the higher mortality. In the current study, the NLR was remained below 11.75 in most of the cured patients (Figure S3G) although it fluctuated greatly at higher levels in the fatal patients (Figure S3H). In the control group, the NLR fluctuated until 14–28 days postdisease onset (Figure S3I).

The right timing for antibody therapy is still unclear. We hypothesized that early treatment would favor the recipient to initialize robust antibody response by themselves. Patient S1, S5, S6, and S7 started therapy at 12, 8, 7, and 7 days after the symptoms onset, respectively. They showed increased neutralizing antibody titers along with the increased IgG, IgM, IgA, and total immmunoglobulin concentrations after the concentrated antibody treatment (Figure 2A, B). In contrast, patient S3 and S4 received the concentrated antibody therapy at 29 and 21 days after the symptoms onset, respectively. Little improvement in either neutralizing or total antibodies was observed by the treatment in the two patients (Figure 2A, B). Patient S2 received the treatment at 13 days after the symptom onset. The total immmunoglobulin and neutralizing antibody levels reach stable high level after the antibody transfusion (Figure 2B). These preliminary data indicated that the earlier Ab treatment possibly facilitated disease recovery from severe COVID‐19.

**FIGURE 2:**
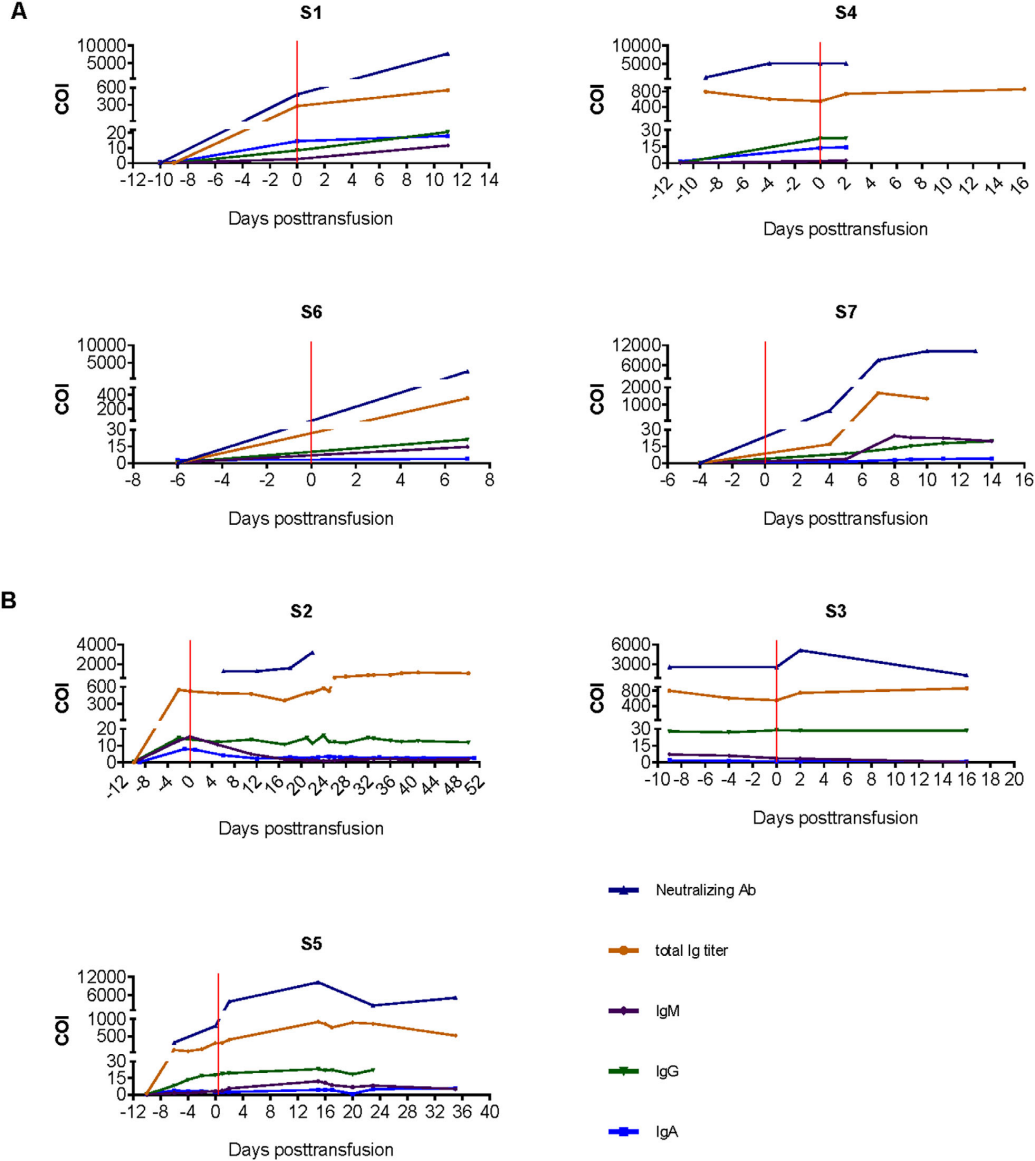
Monitoring binding antibody and neutralizing antibody levels among the treated COVID‐19 patients. The levels of neutralizing antibody and RBD‐binding antibody (IgG, IgM, IgA, and total Ig) before and after concentrated antibody therapy in treated patients S1–S7. (A) Cured patients (S1, S4, S6, S7); (B) fatal patients (S2, S3, S5)

We suspect that the antibody therapy may also affect proinflammatory responses because antibody‐dependent cytokine release (ADCR). Plasma IL‐6, procalcitonin, and C‐reacting protein (CRP) were dynamically monitored. After antibody treatment, the IL‐6 levels were increased rapidly in six of the seven patients (Figure S4A). Notably, IL‐6 in patient S2 remained at a lower level for 30 days after the first antibody treatment, but showed a transient increase after the second antibody treatment then followed by decrease to low levels (Figure S4B). We also observed the similar changes of CRP, procalcitonin mirrored IL‐6 patterns in patient S1 to S7 (Figure S4C–F).

In summary, we found that concentrated antibody therapy may help clear viral reservoirs in infected lung tissues of critical COVID‐19 patients, likely through antibody mediated effector functions. Although the transfused antibodies may cause a transient increase of the inflammatory cytokines, they contribute to the improvement of the overall immune homeostasis and could be used during the early phase of COVID‐19. These findings provide novel evidences for ongoing monoclonal antibody therapy for COVID‐19.

## FUNDING INFORMATION

This study was supported by the National Science Fund for Distinguished Young Scholars (82025022), the Science and Technology Innovation Committee of Shenzhen Municipality (JSGG20200207155251653, JSGG20200807171401008, KQTD20200909113758004), and the Guangdong Basic Applied Basic Research Foundation (2020B111122002 and 2020B1111340035).

## CONFLICT OF INTEREST

The authors declare that there is no conflict of interest.

## Supporting information

Supporting Information

Supporting Information

Supporting Information

Supporting Information

Supporting Information

Supporting Information

Supporting Information
