# Supplementary material for: The concentrated antibody from convalescent plasma balanced the dysfunctional immune responses in patients with critical COVID‐19
Source: Clin Transl Med. 2021 Nov 4;11(11):e571. doi: 10.1002/ctm2.571 (PMC8567045; doi:10.1002/ctm2.571)
Supplement: Supplementary file 1 — Supporting Information [file CTM2-11-e571-s004.docx]

**Methods and Materials**

*Study design and participants*

This was a prospective, non-randomized intervention trial due to the lack of convalescent plasma in the early stages of the COVID-19 outbreak. Patients who were infected during the early stages of the epidemic were considered clinically urgent and thus received a combination of convalescent plasma treatment, as the preparation of a concentrated antibody procedure was relatively more complicated than the direct use of convalescent plasma for treatment. Ethical approval was obtained from the Research Ethics Committee of Shenzhen Third People’s Hospital (2020-001-02). All participants provided written, informed consent.

Thirteen COVID-19 patients were enrolled in this study at the Shenzhen Third People’s Hospital from late January to August, 2020. Diagnosis of SARS-CoV-2 was confirmed by SARS-CoV-2 RNA commercial RT-qPCR assays. All COVID-19 patients were critically ill (receiving mechanical ventilation) according to the “Diagnosis and Treatment Protocol of COVID-19 (the 8th Tentative Version)” issued by National Health Commission of China and to the WHO severity classification. The medical data of the participants was described in Table 1. Patient demographic data, including COVID-19 disease severity, time of disease onset, age, and epidemiological history were recorded. A summary of the data can be found in Table 1. Seven patients (S1-S7) received concentrated antibody treatment as the intervention group (including three who received both convalescent plasma and concentrated antibody). The intervention group patient received 1.2 grams (Patient S1 and the first treatment of S2) and 2.5 grams (the second treatment of S2 and S3-S7) of convalescent plasma concentrated antibody intravenously for two consecutive days. Antibody was administered between 7 to 47 days post symptom onset. And the other six patients (S8-S13) receiving no plasma or antibody therapy were followed as the control group. Patients in the control group had similar baseline clinical characteristics with the intervention group, including age, disease severity, time after symptom onset, and epidemiological history, etc. The inclusion criteria for this study included COVID-19 critically ill patients. Patients exclusion criteria included: 1) definite diagnosis of an incurable malignancy; 2) history of severe cardiovascular or cerebrovascular disease; 3) being on medications with hepatotoxicity; 4) women who were pregnant or lactating, or had recently planned pregnancies; 5) had used or planned to use a drug product other than the antibody used for this observation within 30 days before the first dose of treatment and during the observation period; 6) had an immune deficiency disease (eg, HIV positive), a primary disease in vital organs, cancer (or precancerous lesions), and diseases that might have an effect on the immune response, chronic medical history such as hepatitis B and other liver and kidney diseases outside of nonalcoholic fatty liver disease; 7) history of allergies; 8) asthma; 9) epilepsy; 10) inability to comply with observation related requirements due to psychological or psychiatric abnormalities.

*Convalescent plasma donors*

Concentrated antibodies were derived from the plasma of fourteen convalescent COVID-19 donors (13 with moderate COVID-19 and 1 with severe COVID-19). Their ages were between 19-54 years. All donors were confirmed cases and were discharged according to the 8^th^ version of “Diagnosis and Treatment Guideline of COVID-19” issued by the National Health Commission of China. All donors were tested to be negative for potential infectious agents, including SARS-CoV-2, hepatitis B, hepatitis C, HIV, syphilis and other respiratory viruses. Serum SARS-CoV-2 specific antibody (RBD-specific IgG/IgM) was confirmed by ELISA. Antibody (IgG) binding titer was greater than 1 : 16200 (end point dilution titer, by enzyme-linked immunosorbent assay [ELISA]) among most donors (Table 2). Convalescent plasma was collected at Shenzhen Blood Center using plasma collection machine (Haemonetics Corporation, MCS^+^, USA). Then the plasma was transported to the biological company for antibody concentration (Weiguang Biological Products, Shenzhen, China). Mixed concentrated antibodies derived from donors 1&2 were given to patient S1 and the first treatment of S2; those from donors 3-9 were given to patient S3&S4 and the first treatment of S5; those from donors10-14 were given to patient S6&S7 and the second treatment of S2&S5.

*Main outcomes and measures*

SARS-CoV-2 viral loads, intracellular viral reads, number of peripheral lymphocytes, neutrophil/lymphocyte ratio (NLR), SARS-CoV-2 antibody titers, neutralizing antibody titers, and the level of interleukin (IL)-6, procalcitonin (PCT), and C reactive protein (CRP) in blood were monitored before and after concentrated antibody therapy. BALF cells were collected for single-cell transcriptome sequencing (scRNA-seq) and viral reads were analyzed in patients S1 and S2 at the day when treatment began and 6 days post treatment.

*Convalescent plasma concentrated antibody isolation*

The plasma was thawed and mixed. Then the concentrated antibodies were purified by caprylic acid precipitation, column chromatography and ultrafiltration, and cleaned by low pH incubation and pasteurization for virus inactivation. The quality of concentrated plasma antibody, including protein content, purity, molecular size distribution, kallikrein activator, anti-complement activity, anti-A and anti-B hemagglutinin, HBsAg, Treponema pallidum, HIV-1 and HIV-2 antibodies, and HCV antibodies in the concentrated plasma antibody were determined according to the standards in Chinese Pharmacopoeia (2015 Edition). The bacterial endotoxin was determined according to the European Pharmacopoeia (9th Edition). In addition, the SARS-CoV-2 nucleic acid and antibody titer was confirmed.

*Laboratory measures*

**Viral load** Total RNA was extracted from serial samples collected using the QIAamp RNA Viral Kit (Qiagen, Heiden, Germany) and qRT-PCR was performed using SARS-CoV-2 detection kit (GeneoDX Co., Ltd., Shanghai, China) approved by China Food and Drug Administration (CFDA). Specimens were considered positive if the Ct value was ≤ 37.0. Specimens with a Ct value higher than 37 will be double checked.

**Antibody titers** The blood was centrifuged and 50 μl plasma was examined using the automated chemiluminescence machine (Caris 200, Wantai BioPhar, Beijing), following instructions of the SARS-CoV-2 antibody detection kit (IgA, IgG, IgM and total antibody, innoDx, Xiamen). The ratio of sample to control (COI) was evaluated. The results of COI ≥ 1 are considered positive. The SARS-CoV-2 specific antibody (RBD-specific) IgG/ IgM in donor serum was detected by ELISA assay as described previously^1^.

**Viral infection analysis in Bronchoalveolar lavage fluid (BALF) cells** Isolation of BALF cells, scRNA-Seq library construction and sequencing, scRNA-seq data alignment and sample aggregating, dimensionality reduction and clustering, differential gene expression analysis for clusters were conducted according to previous protocol^2^. To explore the SRAS-CoV-2 and host interaction, Viral-Track algorithm was used to analyze the viral reads in scRNA-seq data with default parameters^3^.

**Serum Neutralization Assay** This was finished in BSL-3 laboratory. Assay was performed as described previously^1^.

**Laboratory testing** Blood cells were examined by Hematology Analyzer (Sysmex XN2000, Japan). PCT (Cat# 5056888200) and IL-6 (Cat# 5109442) were examined using Cobas 6000 (Roche); blood biochemical markers were analyzed by Automatic biochemical analyzer (ADVIA2400, Siemens, USA); and CRP was examined using Diasys reagents (Germany, LOT 60135043). Peripheral lymphocytes counts were examined following instructions of the detection kit (BD Multitest™CD3/CD8/CD45/CD4, 342417) and analyzed by BD FACS Canto II.

*Data availability*

All data used in this study, including scRNA-seq raw data and the expression matrix that supported the findings of this study will be released via a material transfer agreement upon reasonable request. The source code and software pipeline to reproduce the analyses can also be assessed upon request.

*Statistical analysis*

Graphpad prism 8.0.1 software was used for statistical analysis. Tukey's multiple comparison test in one-way ANOVA was used for the change of viral load before and after antibody treatment. P value less than 0.05 indicates significant difference and p value greater than or equal to 0.05 indicates no statistical difference. Chi-square test or Fisher's exact test was used for categorical variables, and Student t test was used for continuous variables.

**REFERENCES**

1. Shen C, Wang Z, Zhao F*, et al.* Treatment of 5 Critically Ill Patients With COVID-19 With Convalescent Plasma. *JAMA* 2020; **323**: 1582-1589.

2. Liao M, Liu Y, Yuan J*, et al.* Single-cell landscape of bronchoalveolar immune cells in patients with COVID-19. *Nat Med* 2020; **26**: 842-844.

3. Bost P, Giladi A, Liu Y*, et al.* Host-Viral Infection Maps Reveal Signatures of Severe COVID-19 Patients. *Cell* 2020; **181**: 1475-1488 e1412.
