# Supplementary material for: The concentrated antibody from convalescent plasma balanced the dysfunctional immune responses in patients with critical COVID‐19
Source: Clin Transl Med. 2021 Nov 4;11(11):e571. doi: 10.1002/ctm2.571 (PMC8567045; doi:10.1002/ctm2.571)
Supplement: Supplementary file 2 — Supporting Information [file CTM2-11-e571-s005.docx]

**Supplemental Figure legends**

**Figure S1. Monitoring SARS-CoV-2 viral loads among the enrolled COVID-19 patients.** (A) The serial viral cycle threshold (Ct) values in concentrated antibody therapy in control group. (B) A time course of concentrated antibody treatment and SARS-CoV-2 viral loads. One-way ANOVA was used for the change of viral load before and after antibody treatment in intervention group. 1, The most recent viral loads before treatment; 2-8, 7 consecutive viral loads after antibody treatment for each individual. (C) UMAP projection of SARS-CoV-2 UMIs in the scRNA-seq data. Color scale indicates normalized UMIs calculated by LogNormalize method in Seurat. D0 is the symptom onset date in (A). The square cross, indicates the reception of the convalescent plasma transfusion and the diamond plus indicates the reception of concentrated neutralizing antibody treatment as indicated.

**Figure S2.** Analysis of the T cells in BALFs of COVID-19 patients before and after treatment. (A) The gene expression plot shows the selected DEGs of T cells from post vs. pre-treatment COVID-19 patients. A gene is considered significant with adjust p < 0.05. (B) The GO BP enrichment analysis of DEGs of T cells between post vs. pre-treatment COVID-19 patients shows enriched pathways (adjust p < 0.05).

**Figure S3. Monitoring peripheral lymphocyte counts and Neutrophil /Lymphocyte ratio (NLR) among the enrolled COVID-19 patients.** CD3^+^ lymphocytes (A), CD4^+^ lymphocytes (B), CD8^+^ lymphocytes (C), CD8/CD4 ratio (D, E) and NLR (G, H) before and after concentrated antibody therapy. CD8/CD4 ratio (F) and NLR (I) from the control group. D0 is the date starting antibody transfusion in (A-C). D0 is symptom onset date in (D-I). The red arrow indicates the reception of the second dose of antibody treatment. The blue arrow indicates the reception of a convalescent plasma transfusion.

# Figure S4. Monitoring serum IL-6/CRP/PCT levels among the treated COVID-19 patients. The levels of IL-6 (A, B), CRP (C, D) and PCT (E, F) before and after concentrated antibody therapy. D0 is the date starting the antibody therapy. The red arrow indicates the reception of another dose of antibody treatment. The blue arrow indicates the reception of a convalescent plasma transfusion.
