# Supplementary figures and images for: The concentrated antibody from convalescent plasma balanced the dysfunctional immune responses in patients with critical COVID‐19
Source: Clin Transl Med. 2021 Nov 4;11(11):e571. doi: 10.1002/ctm2.571 (PMC8567045; doi:10.1002/ctm2.571)

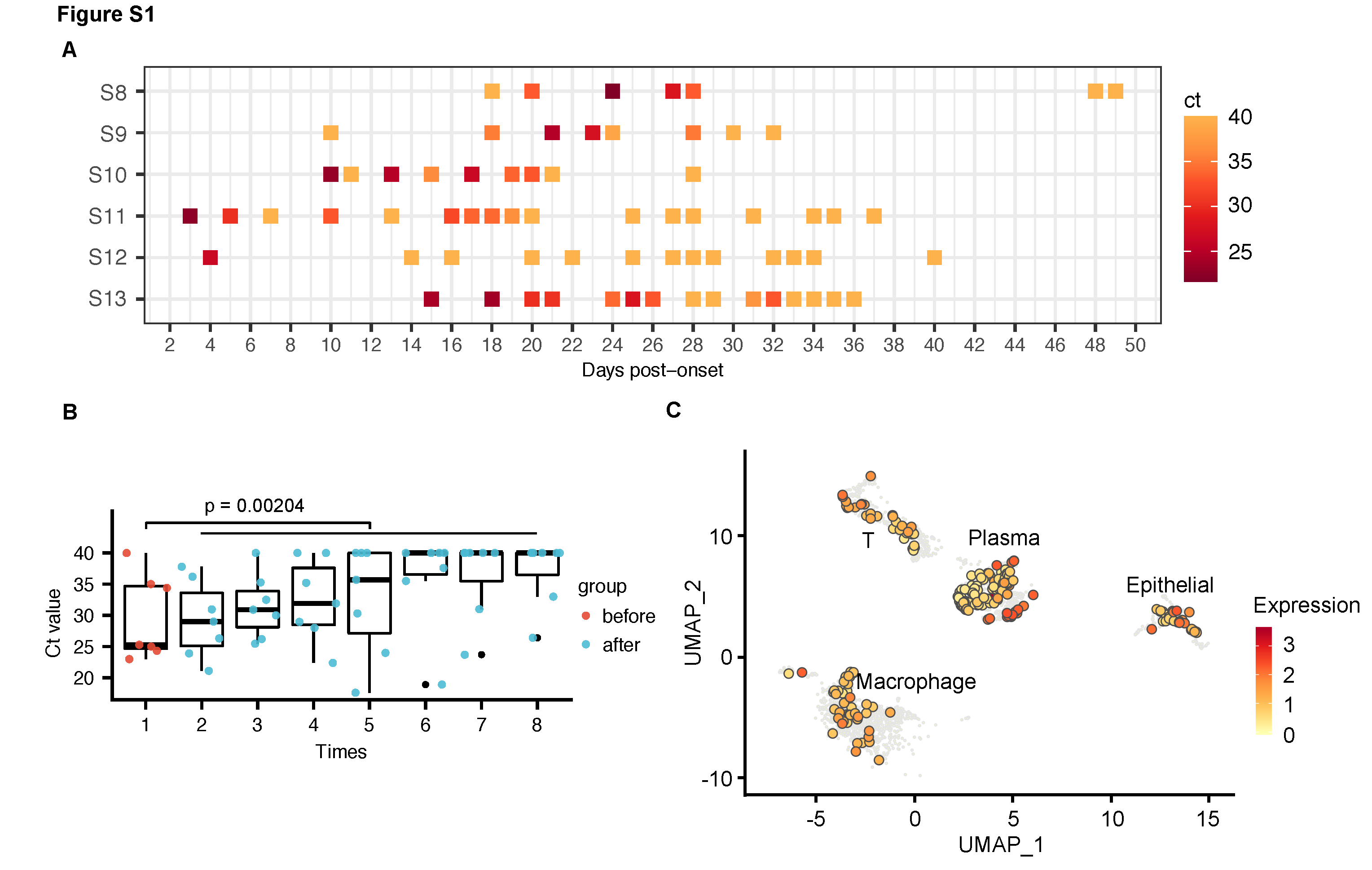

Supplement: Supplementary file 3 — Supporting Information [file CTM2-11-e571-s007.tif]

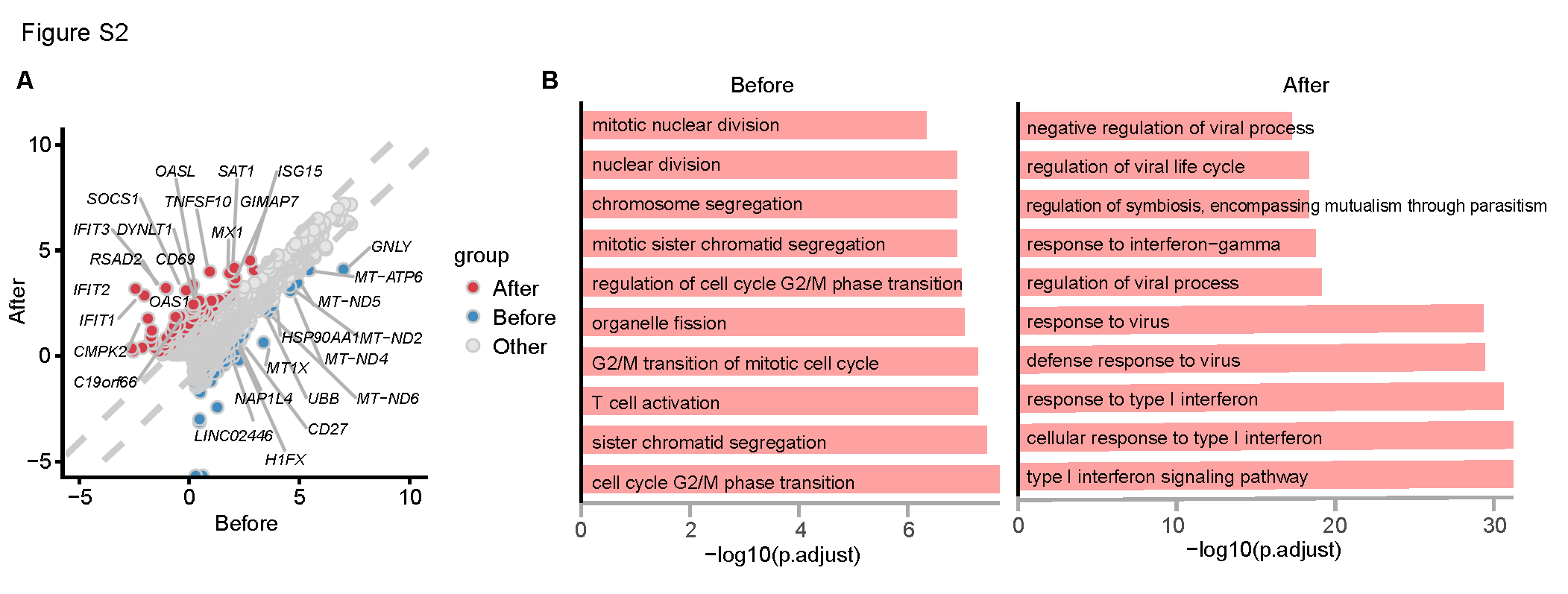

Supplement: Supplementary file 4 — Supporting Information [file CTM2-11-e571-s003.tif]

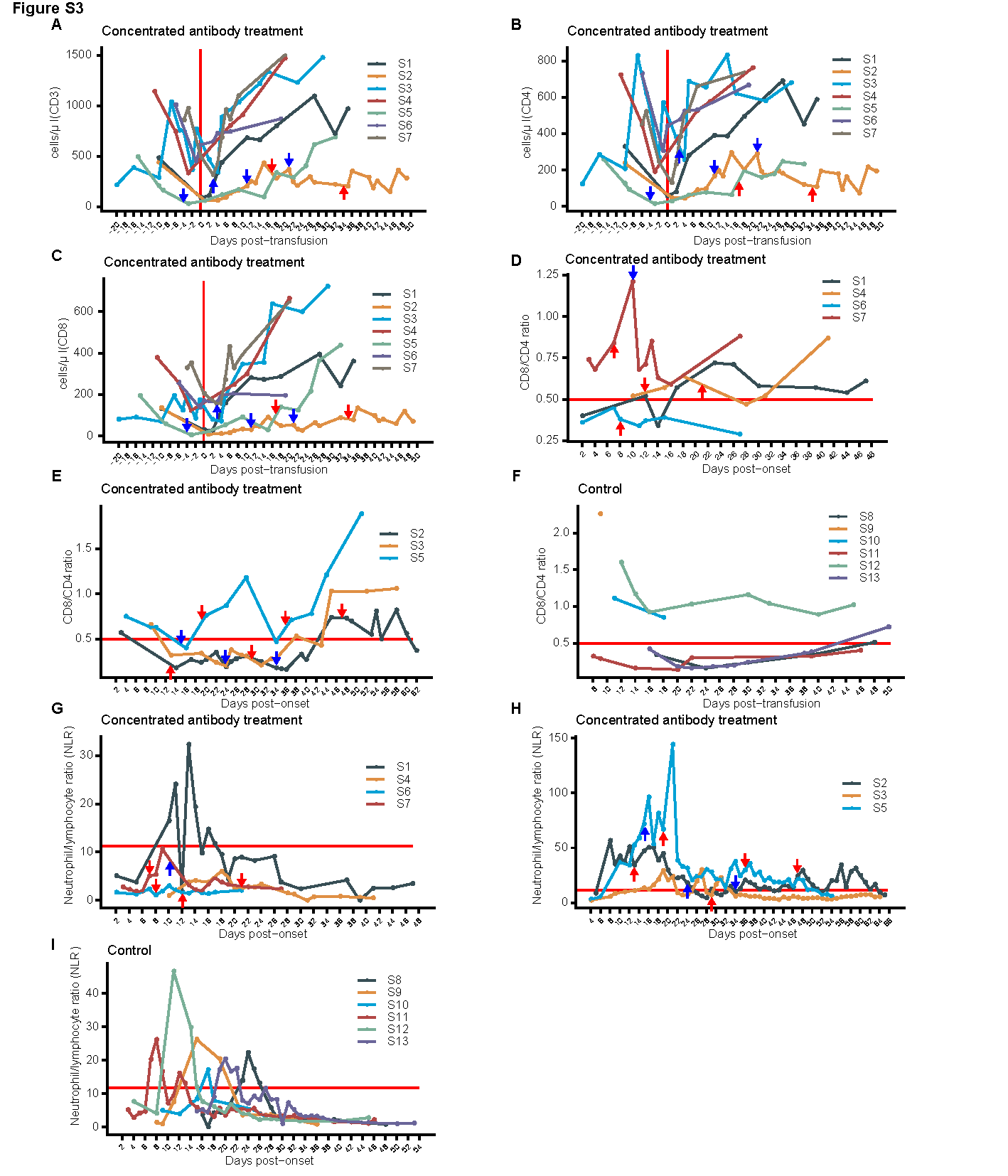

Supplement: Supplementary file 5 — Supporting Information [file CTM2-11-e571-s006.tif]

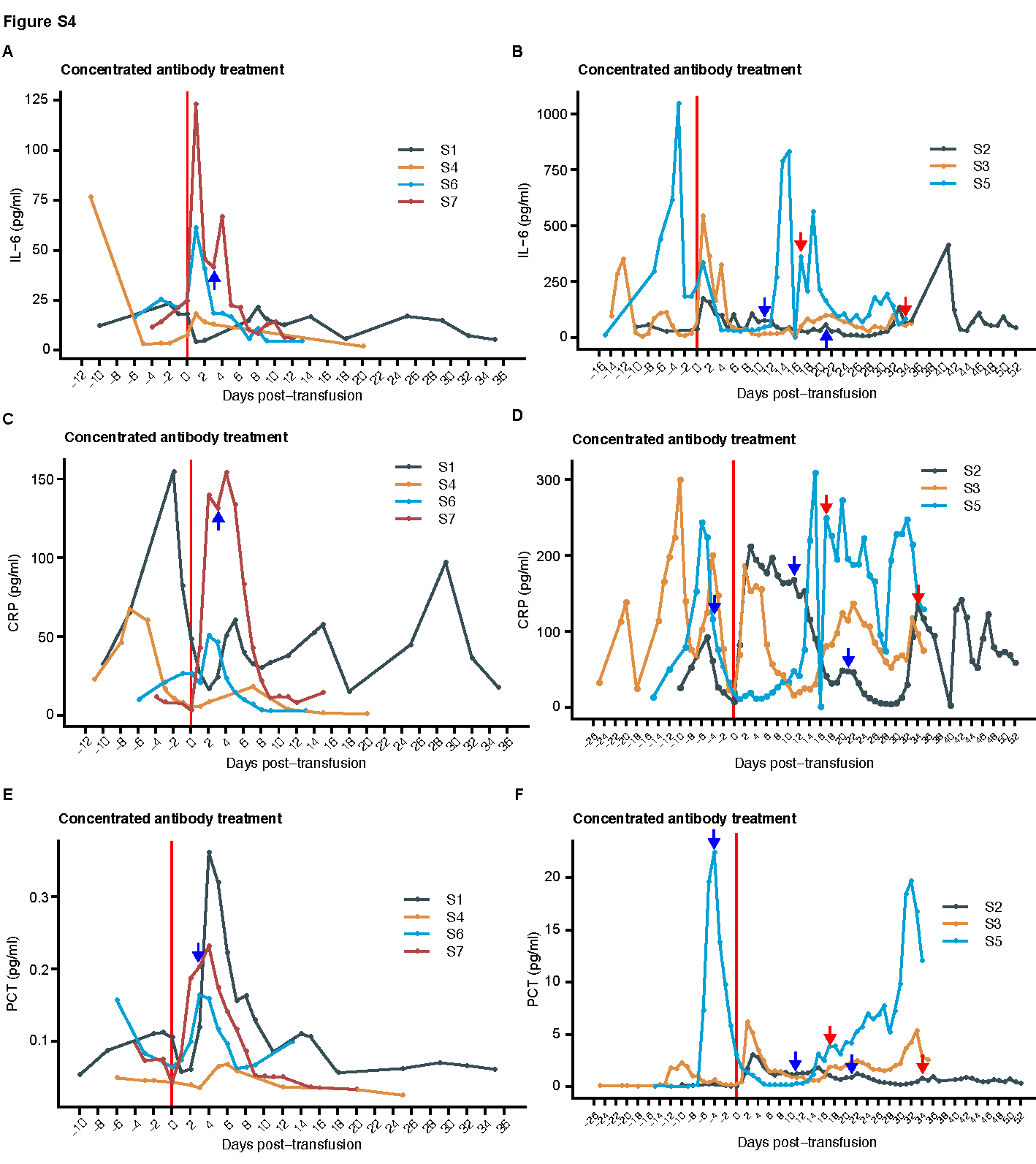

Supplement: Supplementary file 6 — Supporting Information [file CTM2-11-e571-s001.tif]
