# Supplementary material for: The concentrated antibody from convalescent plasma balanced the dysfunctional immune responses in patients with critical COVID‐19
Source: Clin Transl Med. 2021 Nov 4;11(11):e571. doi: 10.1002/ctm2.571 (PMC8567045; doi:10.1002/ctm2.571)
Supplement: Supplementary file 7 — Supporting Information [file CTM2-11-e571-s002.docx]

**Supplemental table 1. Comparison of health history of the control group and intervention group**

|  | level | Overall | Control | Intervention | p value |
| --- | --- | --- | --- | --- | --- |
| n |  | 13 | 6 | 7 |  |
| Severity (%) | Critical | 13 (100.0) | 6 (100.0) | 7 (100.0) | NA |
| Age (years) (mean (SD)) |  | 60.23 (14.31) | 60.50 (13.07) | 60.00 (16.35) | 0.953 |
| Gender (%) | Female | 4 (30.8) | 2 (33.3) | 2 (28.6) | 1 |
|  | Male | 9 (69.2) | 4 (66.7) | 5 (71.4) |  |
| Wuhan travling (%) | No | 8 (61.5) | 4 (66.7) | 4 (57.1) | 1 |
|  | Yes | 5 (38.5) | 2 (33.3) | 3 (42.9) |  |
| Convalescent Plasma Transfusion (%) | ND | 10 (76.9) | 6 (100.0) | 4 (57.1) | 0.243 |
|  | Yes | 3 (23.1) | 0 (0.0) | 3 (42.9) |  |
| Outcome (%) | Cured | 10 (76.9) | 6 (100.0) | 4 (57.1) | 0.243 |
|  | Death | 3 (23.1) | 0 (0.0) | 3 (42.9) |  |
| Chronic basic disease (%) | None | 5 (38.5) | 4 (66.7) | 1 (14.3) | 0.173 |
|  | Yes | 8 (61.5) | 2 (33.3) | 6 (85.7) |  |
| Medication history (%) | None | 13 (100.0) | 6 (100.0) | 7 (100.0) | NA |
| Flu A/B (%) | -/- | 13 (100.0) | 6 (100.0) | 7 (100.0) | NA |
| RSV virus (%) | - | 13 (100.0) | 6 (100.0) | 7 (100.0) | NA |
| Adenovirus (%) | - | 13 (100.0) | 6 (100.0) | 7 (100.0) | NA |
| Methylprednisolone (%) | No | 2 (15.4) | 1 (16.7) | 1 (14.3) | 1 |
|  | Yes | 11 (84.6) | 5 (83.3) | 6 (85.7) |  |
